# Echoes of the Month

**Published:** 1923-07

**Authors:** 


### ECHOES OF THE MONTH.

### MORE DOCTORS THAN EVER.

The rise in the number of registered medical students from 1,875 in the year 1916 to 3,420 in 1919, was succeeded, said the President of the General Medical Council at the Summer Meeting, by a fall to 1,808 in the year 1921. Last year (1922) the number of entrants rose slightly, notwithstanding the higher educational qualification required by some of the bodies, to 1,833. It is still some 400 above the entries for 1912 and 1913. Possibly it may now fall to a point approaching that of the pre-war years, as a result of the new Regulations which came into force at the beginning of this year. The number of medical practitioners registered in 1922 was 1,984, which is 224 above the number for 1921, hitherto the highest on record. The number of names removed from the Register, by reason of death or otherwise, was 1,045. The result is that the total number of medical practitioners now on the Register is 46,477, which considerably exceeds any previous record.

### FOOD SERVICE IN HOSPITALS.

In an article in *The Hospital World* (Toronto) on Food Service in Hospitals, the Supervising Dietitian of Montreal General Hospital calls attention to the newest method of food service which is by heated or insulated conveyors. " These are sent either to ward kitchen or directly to the ward for meal service. Hospitals which have this method are quite enthusiastic in its praises. Certainly the conveyor which takes the food directly to the patient has solved a big hospital problem. These conveyors are either constructed on the fireless-cooker principle, which retains the heat for a long time, or they are electrically heated. The carts having the separately built and insulated compartments are particularly valuable. Heat is retained longer, odours do not mix, and hot and cold foods may be sent by same wagon. As this conveyor may be wheeled around the ward it is never necessary to serve any patient any food which he will not eat. Surely this is much better than serving every plate for every patient, on similar diet orders exactly the same, regardless of idiosyncrasies of different individuals."

### THE PLIGHT OF GERMAN CHILDREN.

One-sixth of all the children's homes and institutions in Germany, according to the *Catholic Times*, have been compelled to close their doors for want of means, expenses being too high. Almost all the children are anæmic and underfed, and their physical and mental development is arrested in the most alarming way. Dr. Davidson, of Halle University, gives the following statistics which he has very carefully tested: " The proportion of children," he says, " infected with tuberculosis is 35 per cent. of those two years old and rises to 67 per cent. for those of six years, so that among children of two every third is infected with this disease." Peofessor Engel, of Dortmund, states: " Our statistics show that over 20 per cent. of the children of our poor at the age of three to five years are unable to walk in consequence of underfeeding and sickness. At Duisburg, again, 6,000 children are reported at the Medical Clinic for Tuberculosis infected with this horrible disease, and at Cleves the deaths from tuberculosis were trebled in 1921. In the whole of Germany there are about six million children infected with tuberculosis."

### CO-OPERATIVE DOCTORS.

An interesting departure in medical treatment is the establishment at Brook Street, W., of a group clinic, an institution which has for some years existed in the United States. " The idea in this country," says " The Scotsman," " was initiated by Dr. Drury Pennington, a general practitioner, and it received support from a number of consultants, including Sir Thomas Horder, who publicly outlined the scheme in an address to the Abernethian Society. The idea is to gather under one roof a group of specialists in different branches of medicine and surgery, together with the lastest equipment for pathological and radiographic inquiry demanded by modern methods of diagnosis. Up to the present the London group numbers fourteen, each member having his own consulting room, with dressing-room attached. After a preliminary examination by the general practitioner, the patient is examined by such consultants as in his opinion are necessary, and a flat rate is charged for the whole inquiry. Thus the patient, it is claimed, will be saved time, money and worry, without any detrimental effect on the income of the individual members of the group.

### VOLUNTARY EUGENICS IN VIENNA.

In Vienna there has been started a " Marriage Advice Bureau," where those contemplating matrimony can seek free expert medical advice. The bureau is conducted on an entirely voluntary basis. In each case heredity and personal medical history have to be considered. It is true that our knowledge of the working of the laws of heredity is still in its infancy, but by comparisons, statistics, and deductions the specialist can now fairly determine the chances in favour of healthy offspring. The main diseases with which the experts have to deal, apart from congenital organic defects, are tuberculosis, venereal diseases, and mental affections. Then there are cases where a course of treatment is advisable before marriage can be justified. Although the bureau has been in existence for only half a year, the demands on it have been so extensive that the hours of consultation have already had to be doubled.

### SALT AS A REMEDY FOR MINERS' CRAMP.

Speaking to a " Morning Post " representative with reference to Professor Moss's address at the Royal Society on miners' cramp, Sir Charles Sherrington pointed out that some months might have to elapse before any very definite conclusions would be possible. The statement by Mr. Moss that miners' cramp was preventible contained at all events a very valuable suggestion. The conditions under which miners' cramp occurred were those which entailed a very considerable loss of water and salt from the body, through perspiration being extreme. In the high temperatures in some of the mines in Lancashire, for example, a loss of three pounds in weight per hour was quite an average, and men who had to keep that up for seven hours at a stretch could only continue by drinking approximately that amount of water. The present position was that the taking of a small quantity of salt—so little as to be quite tasteless—in water would seem to relieve and protect the men against the troubles which resulted from loss of salt.

### AMERICAN HEALTH FILMS.

The American National Health Council has prepared and published a comprehensive list of educational motion pictures on health subjects. Over 300 titles are included in the list which gives in addition to the title the number of reels or the length; the name of the distributor, the rental or sale price, or both; and a brief note about the subject matter of the film. The titles are classified under child hygiene, personal hygiene, public hygiene, communicable diseases, other diseases, nursing, anatomy, and physiology, &c., and miscellaneous. In addition to the collection of information concerning health films the National Health Council, through the health films committee, is making a study of existing films and is encouraging in numerous ways the production of better health films.

### THE COLOUR OF STRYCHNINE.

The General Medical Council has decided against the proposal to colour strychnine a brilliant green. It was held that the question of distinguishing certain poisons by means of artificial coloration merited consideration in connection with the revision of the Pharmacopœia, but should not be summarily determined, as proposed, in the case of a single preparation of poison. It was felt, moreover, that the result of such a practice would be that dispensers might have to keep a stock of uncoloured solutions side by side with the stock of coloured poison, and the risk of confusion and mistake would be correspondingly increased. The fact of British strychnine being strongly coloured while the corresponding official solutions of other national pharmacopœias were uncoloured might give rise to difficulties of an international kind.

